# Acoustic frequency combs using gas bubble cluster oscillations in liquids: a proof of concept

**DOI:** 10.1038/s41598-020-79567-6

**Published:** 2021-01-08

**Authors:** Bui Quoc Huy Nguyen, Ivan S. Maksymov, Sergey A. Suslov

**Affiliations:** 1 Optical Sciences Centre, Swinburne University of Technology, Hawthorn, VIC 3122 Australia; 2 Department of Mathematics, Swinburne University of Technology, Hawthorn, VIC 3122 Australia

**Keywords:** Physics, Applied physics, Biological physics, Fluid dynamics

## Abstract

We propose a new approach to the generation of acoustic frequency combs (AFC)—signals with spectra containing equidistant coherent peaks. AFCs are essential for a number of sensing and measurement applications, where the established technology of optical frequency combs suffers from fundamental physical limitations. Our proof-of-principle experiments demonstrate that nonlinear oscillations of a gas bubble cluster in water insonated by a low-pressure single-frequency ultrasound wave produce signals with spectra consisting of equally spaced peaks originating from the interaction of the driving ultrasound wave with the response of the bubble cluster at its natural frequency. The so-generated AFC posses essential characteristics of optical frequency combs and thus, similar to their optical counterparts, can be used to measure various physical, chemical and biological quantities.

## Introduction

Optical frequency combs—optical spectra composed of equidistant narrow peaks—enable precision measurement in both fundamental and applied contexts^1–6^. An optical frequency comb acts as a spectrum synthesizer that enables the precise transfer of phase and frequency information from a stabilised reference to optical signals. The so-generated signals can be used, for example, to obtain the spectral response of a gas or liquid sample due to linear or nonlinear absorption of light by the medium^7^. One can also accurately measure distances by passing an optical frequency comb signal through an interferometer and then analysing the resulting interference pattern, which is beneficial for the fields of satellite positioning and material science^8^.

**Figure 1:**
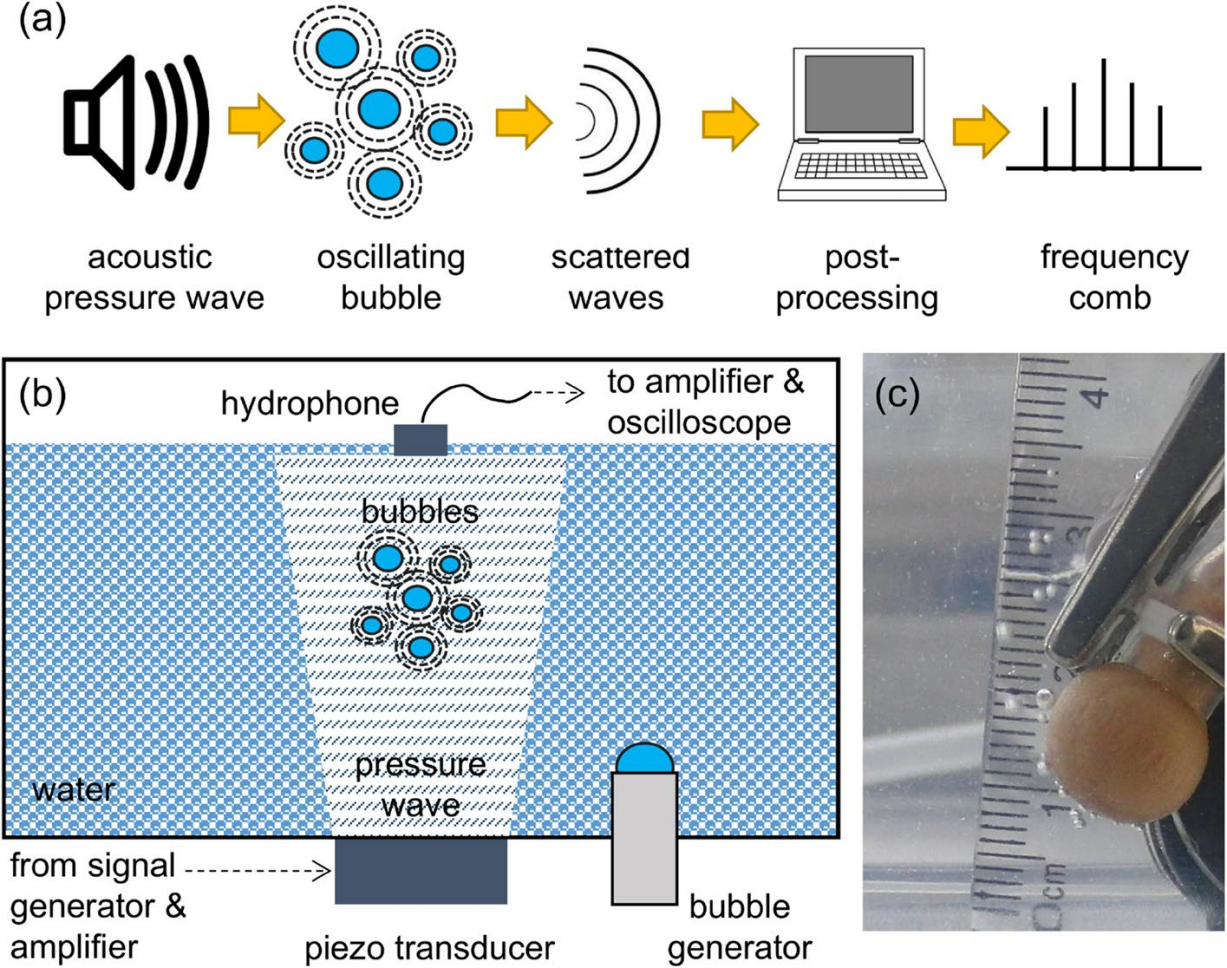
**(a)** Schematic diagram of the suggested AFC generation. The oscillations of the bubble cluster are driven by a single-frequency ultrasound pressure wave. Acoustic waves scattered by the bubble cluster are recorded and post-processed to obtain a spectrum consisting of the equidistant peaks. **(b)** Schematic of the experimental setup. Bubbles are created in a stainless steel tank using a bubble generator. The driving pressure wave is emitted by an ultrasonic transducer. Waves scattered by the bubble are detected by a hydrophone. bf (c) Photograph of typical gas bubbles emitted by the bubble generator in a water tank with transparent walls at otherwise identical experimental conditions to those in the stainless steel tank. The diffuser of the bubble generator and other elements of the setup can be seen.

However, using optical frequency combs is not always possible because of a number of fundamental and technical limitations. For example, in liquid samples such as biological fluids light can be strongly reflected and absorbed by the medium. Photoacoustic frequency comb spectroscopy may help to partially resolve these problems^9,10^, because this technique exploits absorption of light and concomitant generation of acoustic waves that carry information about the absorption strength. Yet, more versatile and technologically simple approaches are still required.

Similar to optical frequency combs, acoustic (phononic) frequency combs (AFC)—purely acoustic signals with spectra containing equidistant coherent peaks—exploit the ability of acoustic waves to provide precision information about the medium in which they propagate^11–17^. In contrast to light, acoustic waves can propagate in water and opaque liquids over long distances, which underpins many acoustics-based technologies including sonar, underwater communication and sensing^15^ and marine biology^18^. Yet, even though AFCs have already been used to accurately measure distances between underwater objects^15^, research on them remains under-established. The development of new types of acoustic combs is needed for sensing and imaging systems^11,14^, in particular, biomedical imaging^16,19,20^.

In this work, we demonstrate the possibility of the AFC generation using a gas bubble cluster nonlinearly oscillating in water^21^, when it is driven by a single-frequency ultrasound wave (Fig. 1a). Unlike in the scenario of an optical frequency comb generation using a high-power laser light and exploiting fundamentally weak nonlinear-optical effects^16^, we show that the application of low-pressure harmonic signals can trigger a strong nonlinear response of the cluster resulting in the generation of multiple ultraharmonic frequency peaks. The interaction with stochastic noise-induced bubble cluster oscillations at its natural frequency, which is typically much lower than that of a driving ultrasound, results in the amplitude modulation of the the bubble cluster response and the appearance of sidebands around the main peaks. This process is thresholdless and therefore the sidebands can always be experimentally observed when their amplitude is higher than the noise floor.

Our current findings contribute to further development of an emergent field of AFC generation^11,14–17^. They also extend our previous observation of frequency combs originating from the onset of Faraday waves in vertically vibrated liquid drops^22,23^. However, in that system the spacing between the peaks of the comb was only 20–40 Hz. Whereas frequency combs with a Hz-range spacing can find certain applications^17^, in the gas bubble system investigated in the present work we use the high-kHz range that can potentially be extended to the high-MHz range^24^. This opens up opportunities for using acoustic combs instead of optical ones or in addition to them in a number of practical situations where operation at higher frequencies may be required^16^.

## Methods

### Experiment

Our experimental setup shown in Fig. 1b consists of a 1.5 L thin-walled stainless steel tank filled with distilled degassed water maintained at room temperature. A generic piezoceramic disc transducer with the measured resonance frequency of 42.6 ± 0.3 kHz is glued to the bottom wall of the tank from outside. The entire apparatus is assembled on a customised vibration damping support. The laboratory room is located in a single-level standalone building with an approximately 50 cm thick concrete floor. The building is located several kilometres away from railways, highways and building sites, where heavy machinery and equipment are used. Together with electric shielding provided by metalled walls, this ensures that external vibrations and interference with high-voltage equipment do not affect results of our measurements.

The piezo transducer is driven by a digital tone generator (Rigol DG-1022Z, China) connected to a broadband power amplifier (Bosch Plena LBB1906/10, Germany). The signal is fed to the piezo-disc via an electrical impedance-matching circuit featuring a customised adjustable mH-range inductor coil (Scientific, Australia) connected in series with the piezo-ceramic transducer. Effectively, the piezo-ceramic disc behaves as a capacitor that draws little current from the amplifier but requires high voltage that in our case is produced by resonance tuning of the *LC* circuit on the frequency of interest. Hence, in our measurements we fix the frequency of the driving ultrasound wave and change its pressure amplitude because this does not require re-tuning the inductor coil.

The hydrophone is based on a small piezoceramic disc (type PIC155, PICeramic, Germany). Electric signals produced by the piezo disc are first amplified using a broadband voltage amplifier (BWD 603B, Australia) with the frequency response from 0 to 100 kHz. Then the signal is sent to a digital oscilloscope (Rigol, DS-1202ZE, China) controlled via a laptop computer.

We use a customised bubble generator consisting of an air pump connected to a silicone tubing terminating in a diffuser made of a piece of porous material. The generator produces several single bubbles per second with the radius of 1.0 ± 0.5 mm. The size of the bubbles was estimated using high-speed digital video camera records (see Fig. 1c).

The large bubbles rise under the effect of buoyancy. Some of them become trapped in the middle of the water layer due to the primary Bjerknes force of the ultrasound pressure wave^21^. As a result, a cluster of bubbles is formed. We record scattered pressure signals produced by the bubble clusters using the hydrophone and then process the measured time-domain signals in the Octave software to obtain spectral information.

By using high-speed imaging we estimate that on average the radius of a bubble cluster is $R_c=20\pm 5$ mm and the air fraction (the ratio of the total volume of bubbles in a cluster to the volume of a region occupied by the bubble cluster) inside it^25–27^ is $\chi \approx 0.015$. This implies that in our case the product $\sqrt{\chi }R_c$ is larger than the radius of the largest bubble in the cluster. According to Eq. (5), this means that the detected frequency $f_c$ should be smaller than the natural frequency of individual bubbles, which is indeed confirmed by our experimental observations.

### Model

Modelling bubble clusters is a challenging task given that their geometry varies from experiment to experiment and with time. Therefore, models considering a cluster as a single equivalent bubble of the size larger than that of constituent bubbles are frequently used^25,26,28^, especially when of interest is the natural oscillation frequency of the cluster as a whole, which is the case in the current work. However, when doing so, one needs to keep in mind that the ultrasound energy absorption and scattering characteristics of a cluster may differ from those of an equivalent single bubble^25,29^. The scattering ($\sigma _{scat}$) and absorption ($\sigma _{abs}$) cross-sections of a single bubble placed in the field of an incident plane ultrasound wave are defined as the ratios of the scattered and absorbed powers, respectively, to the power of the incident wave^30,31^. The extinction cross-section $\sigma _{ext}=\sigma _{scat}+\sigma _{abs}$ characterises the incident wave energy loss due to its absorption and scattering by the bubble. Generally speaking, a large gas bubble behaves as a strong acoustic scatterer with $\sigma _{scat}$ proportional to the square of the bubble radius^29^. Because $\sigma _{abs}$ also scales with the square of the radius^30,31^, larger bubbles have larger $\sigma _{ext}$. However, the scattering cross-section of a bubble cluster is also proportional to the air fraction inside the cluster: $\sigma _{scat\,c}=\chi R_c^2$. Because in our case $\chi \approx 0.015$, the absorption and scattering by the bubble cluster detected in experiments are smaller than those by a single equivalent bubble.

The excitation of a bubble cluster with a single-frequency signal is also known to result in a stronger nonlinear generation of ultraharmonics^31,32^ compared with the case of a single bubble. This is also observed in our experiments. However, these features are inconsequential in the context of AFC generation, which is the main focus of the current work. Therefore, we rely on the results obtained using an equivalent single bubble model discussed below to explain the experimentally detected acoustic response spectra.

The accepted model of nonlinear oscillations of a single spherical bubble in water is given by the Keller–Miksis (KM) equation^33^. It takes into account the decay of bubble oscillations due to viscous dissipation and fluid compressibility. However, in this work we investigate millimetre-sized gas bubbles in water oscillating at 20–100 kHz frequencies and driven by low pressure waves with amplitude of up to 25 kPa. We established that in this regime the terms of the KM equation accounting for acoustic losses are negligible. Thus, in the following we omit these terms thereby effectively reducing the KM model to the classical Rayleigh-Plesset (RP) equation^34,35^:

1 $$\begin{aligned} R\frac{d^2R}{dt^2}+\frac{3}{2}\left( \frac{dR}{dt}\right) ^2 =\frac{1}{\rho }\left( P\left( R,\frac{dR}{dt}\right) -P_\infty (t)\right) \,,\quad R(0)=R_0+\tilde{R}_0\,,\quad \frac{d}{dt}R(0)=V\,, \end{aligned}$$

where $\tilde{R}$ is the initial deviation of the bubble radius from the equilibrium value $R_0$, *V* is the initial speed of the bubble wall,

2 $$\begin{aligned} P\left( R,\frac{dR}{dt}\right) =\left( P_0-P_v+\frac{2\sigma }{R_0}\right) \left( \frac{R_0}{R}\right) ^{3\kappa } -\frac{4\mu }{R}\frac{dR}{dt}-\frac{2\sigma }{R} \end{aligned}$$

and the expression $P_\infty (t)=P_0-P_v+\alpha \sin (\omega ^* t)$ with $\omega ^*=2\pi f_0$ represents the periodically varied pressure in the liquid far from the bubble. The parameters *R*(*t*), $\mu$, $\rho$, $\kappa$, $\sigma$, $\alpha$, and $f_0$ denote, respectively, the instantaneous bubble radius, the dynamic viscosity and the density of the liquid, the polytropic exponent of a gas entrapped in the bubble, the surface tension of a gas-liquid interface and the amplitude and the frequency of a driving ultrasound wave. The diffusion of the gas through the bubble surface is neglected.

In our model oscillations of the bubble are not affected by fluid compressibility, and we can express the acoustic power scattered by the bubble into the far-field zone as^21^

3 $$\begin{aligned} P_{scat}(R,t) = \frac{\rho R}{h}\left( R\frac{d^2R}{dt^2} +2\left( \frac{dR}{dt}\right) ^2\right) , \end{aligned}$$

where $h\gg R_0$ is the distance from the centre of the bubble. The natural frequency of the bubble is^21^

4 $$\begin{aligned} f_{nat}= & {} \frac{1}{2\pi \sqrt{\rho } R_0}\sqrt{3\kappa \left( P_0-P_v+\frac{2\sigma }{R_0}\right) -\frac{2\sigma }{R_0} -\frac{4\mu ^2}{\rho R_0^2}} \approx f_M\left( 1 +\frac{(3\kappa -1)\sigma }{3\kappa R_0\left( P_0-P_v\right) }\right. \nonumber \\&\left. -{\frac{2\mu ^2}{3\kappa \rho R_0^2\left( P_0-P_v\right) }} \right) \,,\quad f_M=\frac{\sqrt{3\kappa \left( P_0-P_v\right) }}{2\pi R_0\sqrt{\rho }} \end{aligned}$$

where $f_M$ is the well-known Minnaert frequency^36^. We use the following fluid parameters corresponding to water at $20^\circ$ C: $\mu =10^{-3}$ kg m/s, $\sigma =7.25\times 10^{-2}$ N/m, $\rho =10^3$ kg/m$^3$ and $P_v=2330$ Pa. In our computations we take the air pressure in a stationary bubble to be $P_0=10^5$ Pa and the polytropic exponent of air to be $\kappa =4/3$^37,38^. For mm-sized air bubbles in water the surface tension and viscous terms in parenthesis in Eq. (4) are of the order of $10^{-4}$ and $10^{-9}$, respectively. Therefore, both the increase of the bubble natural frequency due to the influence of the surface tension and its decrease due to viscous effects can be neglected. The natural frequency of a bubble cluster is given by

5 $$\begin{aligned} f_c\approx \frac{f_M}{\sqrt{\chi }}\frac{R_0}{R_c}, \end{aligned}$$

where $R_c$ is the radius of the bubble cluster and $\chi$ is the air fraction in the liquid^28^. In our experiments, we established that $\sqrt{\chi }R_c > R_0$.

Equation (1) was solved numerically using an explicit Runge-Kutta method^39^ implemented in a standard subroutine ode45 in the Octave software. The numerical solution was used to obtain the acoustic scattering spectra calculated using Eq. (3). In the solver configurations, the numerical values of the absolute and relative error tolerances were set to machine accuracy. However, to identify the main characteristics of nonlinear bubble oscillations relevant to the AFC generation, we present an asymptotic analysis of Eq. (1) next.

Firstly, Eq. (1) is re-written in the non-dimensional form, where we use the equilibrium radius $R_0$ and $t_0=1/\omega ^*$ as the length and time scales, respectively, to introduce non-dimensional quantities $r=R(t)/R_0$ and $\tau ^*= \omega ^*t$. Substituting these into Eq. (1), we obtain

6 $$\begin{aligned} rr''+\frac{3}{2}(r')^2=\frac{\mathcal{M}+\mathcal{W}}{r^\mathcal{K}}-\mathcal{M}-\frac{\mathcal{W}}{r}-\mathcal{R}\frac{r'}{r} +\mathcal{M}_e\sin \tau ^*\,,\quad r(0)=1+\frac{\tilde{R}_0}{R_0}\,,\quad r'(0)=\frac{V}{\omega ^*R_0}, \end{aligned}$$

where prime denotes differentiation with respect to $\tau ^*$, $\displaystyle \mathcal{M}=\frac{P_0-P_v}{\rho {\omega ^*}^2R^2_0}$, $\displaystyle \mathcal{W}=\frac{2\sigma }{\rho {\omega ^*}^2R^3_0}$, $\displaystyle \mathcal{R}=\frac{4\mu }{\rho {\omega ^*} R^2_0}$, $\displaystyle \mathcal{M}_e=\frac{\alpha }{\rho {\omega ^*}^2R^2_0}$ and $\mathcal{K}=3\kappa$. As discussed in Ref.^40^, parameter $\mathcal{M}$ represents elastic properties of the gas and its compressibility, $\mathcal{W}$ and $\mathcal{R}$ can be treated as inverse Weber and Reynolds numbers, characterising the surface tension and viscous dissipation effects, respectively, and $\mathcal{M}_e$ is the measure of the ultrasound forcing. For the conditions of our experiment $\mathcal{K}=4$ and the maximum values of other parameters do not exceed $\mathcal{M}=1.8\times 10^{-3}$, $\mathcal{W}=1.8\times 10^{-6}$, $\mathcal{R}=1.2\times 10^{-5}$ and $\mathcal{M}_e=2.1\times 10^{-4}$. As shown in Ref.^40^, the attenuation of bubble oscillations due to viscous dissipation is proportional to $\exp (-\mathcal{R}\tau '/2)$ and thus is negligible (consequently, we set $\mathcal{R}=0$ in what follows). This suggests that, in the context of the current study, forced bubble oscillations can be assumed perfectly periodic provided that the driving ultrasound frequency is much higher than any of the bubble resonance frequencies (see below). This enables us to make an analytic progress by employing Poincaré–Lindstedt method, which is algebraically simpler than Bogolyubov–Krylov^41^ or multiple scales^42,43^ methods traditionally used to account for various transient processes in bubble dynamics.

We introduce a stretched time variable $\tau =\omega \tau ^*=\omega \omega ^*t$, where $\omega$ is the non-dimensional frequency of nonlinear bubble oscillations that depends on their amplitude. The estimation of physical parameters given above suggests that ultrasound forcing applied to a bubble is relatively weak and, subsequently, it is not expected to cause large amplitude oscillations of a bubble. Therefore, we look for an asymptotic solution of Eq. (6) in the form

7 $$\begin{aligned} r= & {} 1+\epsilon r_1(\tau )+\epsilon ^2r_2(\tau )+\epsilon ^3r_3(\tau )+\ldots , \end{aligned}$$

8 $$\begin{aligned} \omega= & {} \omega _0+\epsilon \omega _1+\epsilon ^2\omega _2+ \ldots \,, \end{aligned}$$

where $\epsilon$ is a formal parameter introduced to distinguish between various terms in the asymptotic series (it is to be set to unity once the series is developed). We also set $\mathcal{W}=0$ given that, as estimated above, the influence of surface tension in our experimental setup is so weak that it cannot be detected within the measurement accuracy. Introducing such a simplification in the mathematical model does not have any effect on the functional form of solutions either but makes the expressions for various solution coefficients much shorter and easier to interpret. Choosing $\omega _0^2=\mathcal{K}(\mathcal{M}+\mathcal{W})-\mathcal{W}=\mathcal{K}\mathcal{M}$, which corresponds to Minnaert frequency given by Eq. (4), substituting Eqs. (7) and  (8) into Eq. (6) and collecting terms at various orders of $\epsilon$, we obtain the following equations (up to $\mathcal{O}(\epsilon ^3)$)

9 $$\begin{aligned} \ddot{r}_1+r_1= & {} p\sin (\Omega \tau ), \end{aligned}$$

10 $$\begin{aligned} \ddot{r}_2+r_2= & {} \frac{1}{2}(1+\mathcal{K})r_1^2 -\frac{3}{2}\dot{r}_1^2 - r_1\ddot{r}_1 -2\frac{\omega _1}{\omega _0}\ddot{r}_1\,, \end{aligned}$$

11 $$\begin{aligned} \ddot{r}_3+r_3= & {} (1+\mathcal{K})r_1r_2-\frac{1}{3}\left( 1+\frac{1}{2}\mathcal{K}(\mathcal{K}+3)\right) r_1^3 +\frac{3\omega _1\dot{r}_1^2}{\omega _0}+3\dot{r}_1\dot{r}_2 +\left( \frac{\omega _1^2}{\omega _0^2} +\frac{2\omega _2}{\omega _0}\right) \ddot{r}_1 + \ddot{r}_1r_2+\frac{2\omega _1}{\omega _0}r_1\ddot{r}_1, \end{aligned}$$

where dot denotes differentiation with respect to $\tau$ and we set $(\mathcal{M}_e/\omega _0^2)\sin \tau ^*=\epsilon p\sin (\Omega \tau )$, $\Omega =1/\omega \gg 1$. We also write the random initial conditions as $r(0)=1+\epsilon a$ and $\dot{r}(0)=\epsilon b$, where $\epsilon a=\tilde{R}_0/R_0$ and $\epsilon b=(V\Omega )/(\omega ^*R_0)$. Then

12 $$\begin{aligned} r_1(0)=a\,,\ r_2(0)=r_3(0)=0\quad \text{ and }\quad \dot{r}_1(0)=b,\ \dot{r}_2(0)=\dot{r}_3(0)=0. \end{aligned}$$

The solution $r_1(\tau )$ is

13 $$\begin{aligned} r_1=a\cos \tau +(b+D\Omega )\sin \tau -D\sin (\Omega \tau ) =R_1\sin (\tau +\phi _1)-D\sin (\Omega \tau ), \end{aligned}$$

where $\displaystyle D=\frac{p}{\Omega ^2-1}$, $R_1=\sqrt{a^2+(b+D\Omega )^2}$ and $\phi _1=\tan ^{-1}{\dfrac{a}{b+D\Omega }}$. This solution is valid away from the main resonance, that is for $\Omega \ne 1$, which is the case in the considered application since the bubble natural frequency is much smaller than that of a driving ultrasound wave and thus $\Omega \gg 1$. In particular, in our experiments $\Omega \approx 1/\omega _0\approx 11.6$. The relevant physical conclusion that follows from Eq. (13) is that unless the initial conditions are chosen in a very specific way ($a=0$ and $b=-D\omega$), the leading order bubble response will always contain two frequencies: the bubble’s natural frequency and the driving ultrasound frequency. It is conceivable that in very careful experiments with a single bubble the excitation of its oscillations at the natural frequency would not occur, or they could decay due to dissipation if the observation time is sufficiently long. Then the generation of the bubble-based AFC would fail. However, this scenario is improbable when a bubble cluster is used, where neighbouring bubbles acoustically interact with each other so that initial conditions *a* and *b* for each individual bubble remain essentially arbitrary. (Indeed, in our earlier experiments with a single large bubble the number of cases, where the oscillations at the natural frequency were not observed, was statistically insignificant and AFCs have been always observed in our experiments with bubble clusters.)

The second relevant conclusion that solution Eq. (13) offers is that in the non-resonant conditions, which are of interest here, the amplitude of both forced and natural bubble oscillation is proportional to that of the driving field. At the same time, the amplitude of forced oscillations is inversely proportional to the square of the driving frequency, while that of the natural oscillations to its first power. This offers a practical opportunity of controlling the power spectrum of the so-generated AFC by optimising the choice of the $(\alpha ,\omega ')$ characteristics of insonification.

To avoid an aperiodic secular term proportional to $\tau$ in the solution of the second-order equation we must eliminate the last term in the right-hand side of Eq. (10) by setting $\omega _1=0$. Then solving Eq. (10) with $r_1(\tau )$ given by Eq. (13) we obtain

14 $$\begin{aligned} r_2= & {} C+c_1\cos \tau +c_2\sin \tau -A\cos (2\tau )-B\sin (2\tau ) +E\cos (2\Omega \tau )\nonumber \\&+F \cos \left( \Omega \tau \right) \left[ (b+D\Omega )\cos \tau -a\sin \tau \right] +G \sin (\Omega \tau )\left[ a\cos \tau +(b+D\Omega )\sin \tau \right] \nonumber \\= & {} C+R_2\sin \left( \tau +\phi _2\right) -R_3\sin \left( 2\tau +\phi _3\right) +E\cos (2\Omega \tau ) +\frac{a}{2}\left[ (G+F)\sin \left[ (\Omega -1)\tau \right] +(G-F)\sin \left[ (\Omega +1)\tau \right] \right] \nonumber \\&+\frac{b+D\Omega }{2} \left[ (G+F)\cos [(\Omega -1)\tau ]-(G-F)\cos [(\Omega +1)\tau ]\right] , \end{aligned}$$

where

$$\begin{aligned} c_1= & {} A-C-E-F(b+D\Omega )\,,\quad c_2=2B+a(F-G\Omega ),\\ A= & {} \frac{1}{12}[a^2-(b+D\Omega )^2](6+\mathcal{K})\,,\quad B=\frac{a}{6} (b+D\Omega )(6+\mathcal{K}),\\ C= & {} \frac{\mathcal{K}}{4}\left[ a^2+(b+D\Omega )^2+D^2\right] +\frac{D^2}{4}(1-\Omega ^2)\,,\quad E=\frac{D^2}{4}\frac{5\Omega ^2+\mathcal{K}+1}{4\Omega ^2-1},\\ F= & {} -\frac{D}{\Omega }\left( 1-\frac{2\mathcal{K}}{\Omega ^2-4}\right) ,\quad G=D\left( 1+\frac{\mathcal{K}}{\Omega ^2-4}\right) ,\\ R_2= & {} \sqrt{c_1^2+c_2^2},\quad \phi _2=\tan ^{-1}{\dfrac{c_1}{c_2}},\quad R_3=\sqrt{A^2+B^2},\quad \phi _3=\tan ^{-1}{\dfrac{A}{B}}. \end{aligned}$$

A number of relevant conclusions can be made from the expression for $r_2$. Firstly, due to the nonlinearity the second harmonics of both natural (2) and forcing ($2\Omega$) frequencies appear in addition to the fundamental harmonics (with non-dimensional frequencies 1 and $\Omega$) and their amplitudes (coefficients *A*, *B* and *E*) are proportional to the squares of the initial conditions and the forcing amplitude. This means that if the forcing and random noise in the system are small, the magnitudes of the spectral lines at such double frequencies are smaller than those of base frequencies. Secondly, the amplitudes *A* and *B* of double natural frequency components decay as $\Omega ^{-2}$ with the forcing frequency while the $2\Omega$ ultraharmonic decays as $\Omega ^{-4}$, see coefficient *E*. Therefore, at high-frequency forcing the ultraharmonics of forcing decay faster than those of natural frequency and this fact can be used to balance the AFC peaks centred at $n\Omega$, $n=2,3,\ldots$. Thirdly, the time average of the bubble radius deviates from $r=1$: since $\mathcal{K}>1$ coefficient *C* is positively defined reflecting the known fact that a nonlinearly oscillating bubble stays in an inflated state for the most of the oscillation period with short-lasting rapid contraction/restoration stages. Fourthly, the solution coefficients become singular at forcing frequencies $\Omega =1/2$ and 2, which indicates the possibility of ultra- and subharmonic resonances when the current solution would break down. This is not of a concern in the AFC context though because here $\Omega \gg 1$ by design.

The asymptotic expansion procedure can be routinely continued to higher orders revealing the possibility of further ultra- and subharmonic resonances at $\Omega =n$ and $\Omega =1/n$ and amplitude modulations with $\Omega \pm n$, $n=1,2,3,\ldots$. However, here we do not pursue this any further and only note that the elimination of secular terms at order $\epsilon ^3$ requires that

15 $$\begin{aligned} \omega _2= & {} \frac{\omega _0}{8} \left[ D^2\left( \frac{2\mathcal{K}^2}{\Omega _0^2-4}+\mathcal{K}\Omega _0^2+2\right) -\frac{a^2+(b+D\Omega _0)^2}{6}\left( 2\mathcal{K}^2-3\mathcal{K}-6\right) \right] \end{aligned}$$

16 $$\begin{aligned}= & {} \frac{\omega _0}{4} \left[ D^2\left( \frac{16}{\Omega _0^2-4}+2\Omega _0^2+1\right) -\frac{7}{6}\left( a^2+(b+D\Omega _0)^2\right) \right] ,\quad \Omega _0=\frac{1}{\omega _0}. \end{aligned}$$

This expression demonstrates that in the noisy conditions (when *a* and/or *b* are non-zero) the frequency of nonlinear bubble oscillations decreases with the square of the initial conditions (the second term in the brackets of Eq. (16) is negative), which is a well established fact^41–43^. However, the application of high-frequency forcing, when $\Omega \approx \Omega _0=1/\omega _0$ and $D\Omega ^2\approx p\sim\mathcal{M}_e/\omega _0^2$, tends to partially compensate for such a noise-induced frequency reduction (the first term in Eq. (16) is positive for large $\Omega$). In terms of physical parameters the relative variation of the bubble natural oscillation frequency in an ultrasound field in this limit is

17 $$\begin{aligned} \frac{\Delta f}{f_M}\approx \frac{\omega _2}{\omega _0} { \approx \frac{5}{24}\left( \frac{\mathcal{M}_e}{\omega _0}\right) ^2 -\frac{7}{24}\left( a^2+b^2+2b\frac{\mathcal{M}_e}{\omega _0}\right) , \quad \frac{\mathcal{M}_e}{\omega _0}=\frac{\alpha }{\rho \omega _0{\omega ^*}^2R_0^2} =\frac{\alpha }{4\pi ^2\rho f_Mf_0R_0^2}}. \end{aligned}$$

The downshift of natural frequency of the bubble oscillation occurs when the noise level exceeds $\displaystyle (a,b)\gtrsim (\mathcal{M}_e/\omega _0)$, which is $\sim 10^{-3}$ in our experiments (i.e. when the deviation of the bubble radius from the equilibrium value caused by a random noise exceeds 0.1%). In this case, the frequency upshift due to ultrasound forcing is negligible and by measuring the downshift of the bubble’s natural frequency one can use Eq. (17) to estimate the average intensity $a^2+b^2$ of natural bubble oscillations.

Finally, we set $\varepsilon =1$ and write the leading terms in the non-dimensional expression $p_{scat}=(P_{scat}h)/(\alpha R_0)$ for the acoustic power scattered by a bubble (see Eq. (3)) as

18 $$\begin{aligned} {\frac{\mathcal{M}_e}{\omega ^2}p_{scat}}= & {} r\left( r\ddot{r}+2\dot{r}^2\right) \nonumber \\\approx & {} -\left( a+c_1\right) \cos \tau -\left( b+D\Omega +c_2\right) \sin \tau +2\left[ 2A-a^2+(b+D\Omega )^2\right] \cos (2\tau ) +4[B-a(b+D\Omega )]\sin (2\tau )\nonumber \\&+\Omega ^2\left[ D\sin (\Omega \tau ) +2(D^2-2E)\cos (2\Omega \tau )\right] \nonumber \\&+\frac{a}{2}\left[ (2D-G-F)(\Omega -1)^2\sin [(\Omega -1)\tau ] +(2D-G+F)(\Omega +1)^2\sin \left[ (\Omega +1)\tau \right] \right] \nonumber \\&+\frac{1}{2}(b+D\Omega )\left[ (2D-G-F)(\Omega -1)^2\cos [(\Omega -1)\tau )] -(2D-G+F)(\Omega +1)^2\cos [(\Omega +1)\tau ]\right] . \end{aligned}$$

To interpret the structure of acoustic bubble response more clearly avoiding excessive algebraic detail we consider a particular case when noise leads to small expansion of a stationary bubble: $0<a\ll 1$, $b=0$. In the limit of high-frequency excitation $\Omega \gg 1$, $-F\approx (D/\Omega )\ll D$, $G\approx D$ and Eq. (18) becomes

19 $$\begin{aligned} p_{scat}\approx & {} -\omega _0 \left[ \sqrt{1+\frac{a^2\omega _0^2}{\mathcal{M}_e^2}} \sin (\tau +\phi _4) +\frac{\mathcal{K}\mathcal{M}_e}{3\omega _0} \sqrt{1+4\frac{a^2\omega _0^2}{\mathcal{M}_e^2}}\sin (2\tau +\phi _5) \right] +\frac{3}{4}\mathcal{M}_e\cos (2\Omega \tau )\nonumber \\&+\sin (\Omega \tau ) \left[ 1+\frac{\mathcal{M}_e}{\omega _0}\sqrt{1+\frac{a^2\omega _0^2}{\mathcal{M}^2_e}} \sin (\tau +\phi _6)\right] \,, \end{aligned}$$

20 $$\begin{aligned} \phi _4= & {} \tan ^{-1}\left[ \frac{a\omega _0}{\mathcal{M}_e} -\frac{3+4\mathcal{K}}{12}\frac{\mathcal{M}_e}{\omega _0}\right] ,\quad \phi _5=\tan ^{-1}\left[ \frac{a\omega _0}{2\mathcal{M}_e} -\frac{\mathcal{M}_e}{2a\omega _0}\right] \,, \quad \phi _6=\tan ^{-1}\frac{a\omega _0}{\mathcal{M}_e}. \end{aligned}$$

For $a\lesssim 0.1$ the values of $p_{scat}$ obtained using Eq. (19) coincide with those computed using a numerical solution of the full Eq. (6) within a few per cent. The term involving driving signal $\sin (\Omega \tau )$ in Eq. (19) represents an acoustic bubble response after it is processed using a combination of low- and high-pass filters as is done in our experiments. It defines a classical beating pattern that arises on the background of sinusoidal oscillations. (Note that in the absence of random noise ($a=b=0$) the relative depth of the resulting beating modulation given by $\mathcal{M}_e/\omega _0$ is small. However, the condition $a=b=0$ cannot be fulfilled in the case of a bubble cluster, which means that the beating pattern observed in experiments involving a bubble cluster can be much stronger than in the case of a single gas bubble.)

We also note that Eq. (19) is a truncated version of the full expression for the acoustic signal scattered by the bubble that only includes terms up to the second harmonics $(2\tau )$ and $(2\Omega \tau )$. Yet it captures all main features of the acoustic signal structure and shows how AFC is generated around the driving ultrasound frequency $\Omega$. The mechanism of generating AFC around higher harmonics $n\Omega$, $n>1$ is similar and does not need to be discussed separately. We only note that it follows from Eq. (19) that the intensity of spectral peaks of the acoustic response detected at frequencies $n\Omega$ decreases compared to that at $\Omega$ as $\mathcal{M}_e^{n-1}$ and the intensity of the *n*th sideband peak is proportional to $(a^n,b^n)$. In practical terms this means that the overall AFC width determined by the highest detectable ultraharmonic frequency $n\Omega$ can be expanded by increasing the amplitude of driving ultrasound waves.

**Figure 2:**
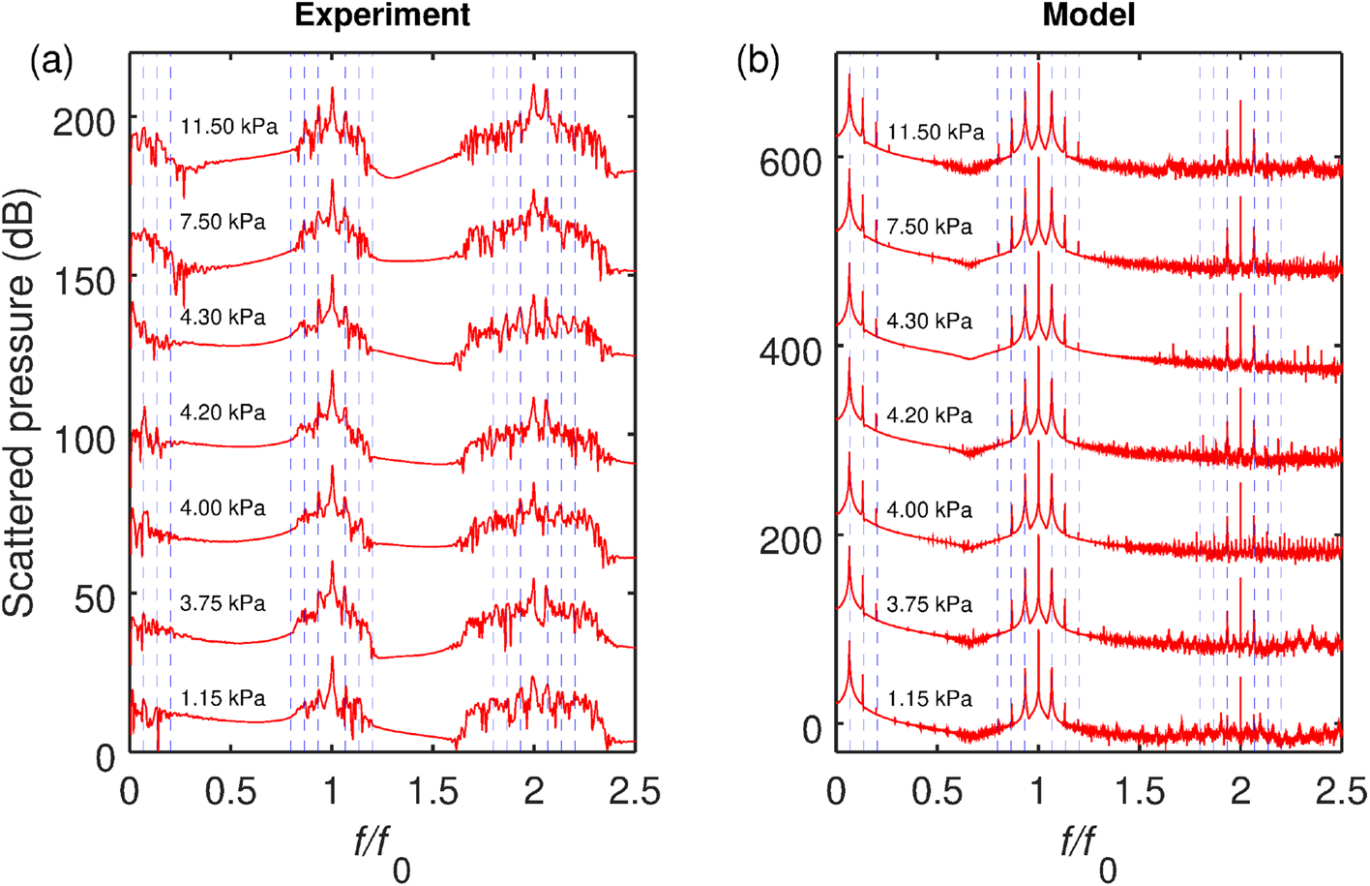
(**a**) Experimental spectra of a cluster of gas bubbles in water insonated with the 24.6 kHz sinusoidal signal of increasing pressure amplitude $\alpha =1.15$, 3.75, 4, 4.2, 4.3, 7.5 and 11.5 kPa. The frequency axis is normalised with frequency $f_0$ of the driving field. The detectable response frequency resolution is $\Delta f/f_0=1.34 \times 10^{-4}$. The scattered pressure values (in dB) are shown along the vertical axis with the vertical offset of 30 dB between spectra. (**b**) Calculated spectra of a single gas bubble with 1.95 mm radius at the same driving pressure frequency and amplitudes as in the experiment. The vertical offset between individual spectra is 100 dB. In both panels, the vertical dashed lines mark the peaks at the natural frequency and its ultraharmonics (the left parts of the spectra) as well as the frequencies of the sideband peaks around the fundamental and second harmonic frequency of the driving signal.

## Results

We demonstrate experimentally fundamental physics behind the principle of the AFC generation, which we suggest, by studying a cluster of bubbles created using a bubble generator. The natural frequency of such a cluster is smaller than that of constituent bubbles because the cluster effectively behaves as a single bubble of radius $R_c > R_0$ (see Eq. 5). This physical similarity also enables us to explain experimental findings by conducting numerical modelling of nonlinear oscillations of a single equivalent spherical bubble in water, see Methods.

Figure 2a shows the measured dependence of the scattering spectrum on the increasing amplitude of the driving pressure $\alpha$ at the driving frequency $f_0=24.6$ kHz. We observe the main features of a frequency comb—a number of equidistant sideband peaks around the fundamental harmonic frequency $f/f_0=1$ marked by the dashed lines. The distance between all sideband peaks is 1.67 kHz, which according to Eq. (5) corresponds to the natural frequency of a bubble cluster with $\sqrt{\chi }R_c=1.95$ mm. The peaks at this natural frequency and its higher-order ultraharmonics are also distinguishable and they are marked by the leftmost dashed line in Fig. 2a.

We also observe that the nonlinearly induced higher-order ultraharmonics of the natural response of the bubble cluster result in the secondary sideband peaks around $f/f_0=1$. In fact, the sideband peaks adjacent to the central peak originate from the natural frequency of the bubble cluster, but the other two are due to the interference with the first ultraharmonic of the cluster response at the twice the natural frequency. A qualitatively similar sideband peak structure can be seen around $f/f_0=2$.

Figure 2b shows the calculated spectra obtained for the experimental values of the frequency and amplitude. Consistently with the size of the bubble cluster inferred from the experiment, in the calculation we assume that the radius of the single equivalent gas bubble is 1.95 mm (significantly, the calculated spectra are qualitatively similar for the bubble radii in the 1–2 mm range). We note an overall good qualitative agreement between the experimental and calculated spectra. In experiments, we can clearly see the primary and secondary sidebands around $f/f_0=1$. The calculation also predicts the existence of the tertiary sidebands at high values of $\alpha$. However, these are undetectable in our measurements due to their low relative magnitude.

The spectra of the experimental response contain components that are not found in calculations even after we remove the random noise floor from raw measured traces. As discussed in section “Methods” we exclude the possibility of a significant external noise due to vibrations and interference with high-voltage equipment. Thus we relate a slight irregularity of the comb lines to the Doppler effect associated with a translational motion of oscillating bubbles in the incident ultrasound field^44,45^. The size variation of the generated bubbles could also contribute to the comb line imperfection. However, we do not consider these deficiencies of initial proof-of-concept experiments as prohibitive for the following reasons. The position of a single oscillating bubble can be stabilised by trapping it in the antinode of an acoustic standing wave field in a resonator^46–48^. Similar techniques can be used to stabilise a cluster of bubbles^49^, which would significantly reduce the random Doppler distortion of acoustic comb spectra. The technical problem with a bubble size variations can also be resolved by using existing higher precision mono-disperse bubble generators (albeit more expensive than that currently available to us)^50,51^.

Interestingly, the tertiary sideband peaks can be seen around $f/f_0=1$ at $\alpha =4.3$ kPa. More broadly, we note a larger magnitude of all experimental peaks at $f/f_0=2$ compared to the calculated values. This observation is consistent with the fact that a response of a bubble cluster rather than of a single bubble is measured: clusters exhibit stronger acoustic nonlinearities^31,32^ that give rise to more energetic signals at the second harmonic frequency $f/f_0=2$.

**Figure 3:**
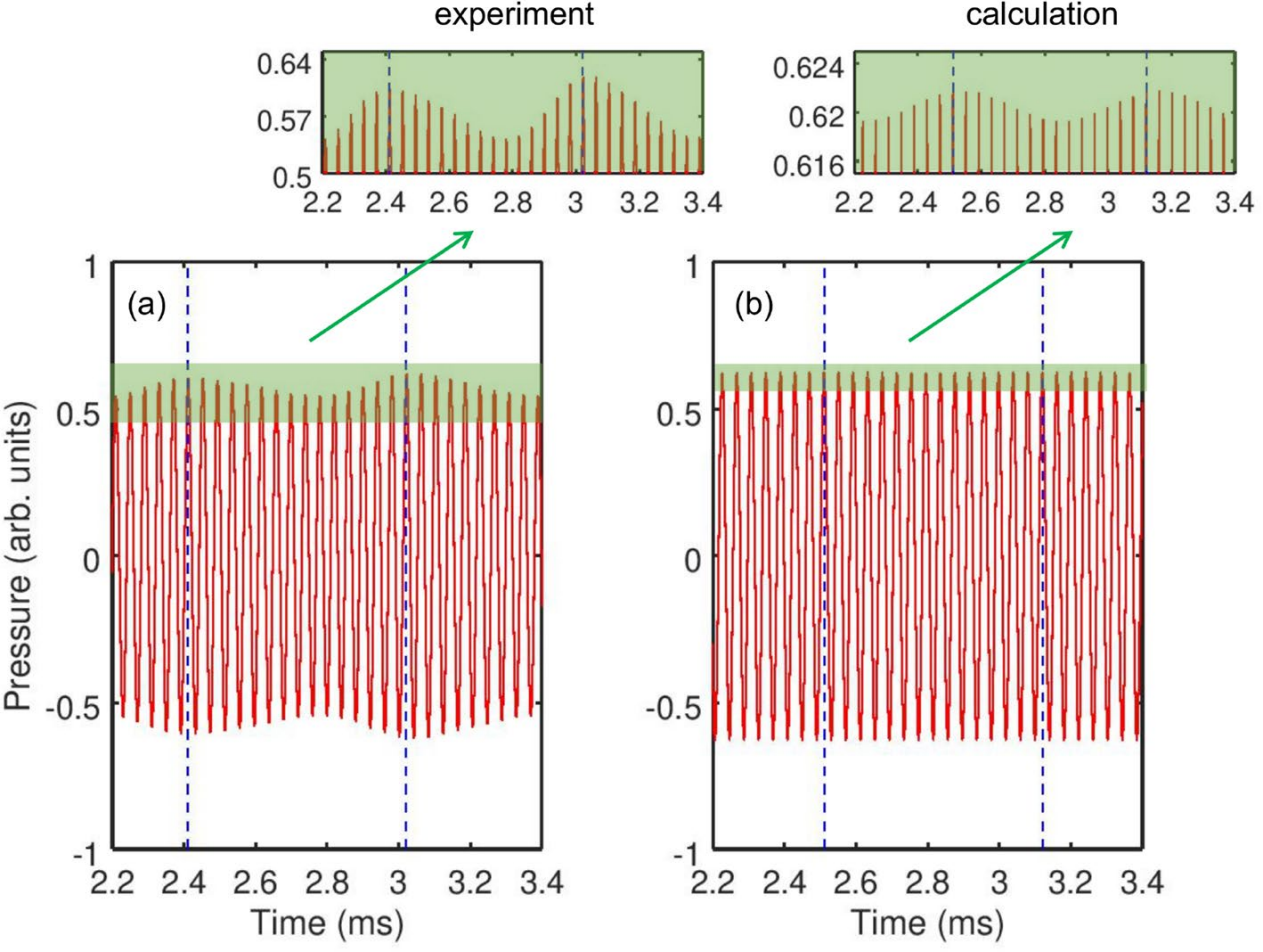
(**a**) Measured acoustic response of the gas bubble cluster and (**b**) calculated acoustic response of a single equivalent bubble corresponding to a sinusoidal pressure wave with the frequency $f_0=24.6$ kHz and amplitude $\alpha =11.5$ kPa. In the calculation, the initial conditions were set to be $R(0)=R_0$ and $\frac{d}{dt}R(0)=0$, see Eq. (1). The time between the vertical dashed lines is $\Delta T=1/f_{nat} \approx 0.6$ ms. The insets show the closeup of the waveforms and demonstrate the amplitude modulation (see the main text for more details.) Arbitrary pressure units are used in both panels to enable the comparison of the experimental and calculated data.

Figure 3a shows the temporal far-field pressure profile corresponding to $f_0=24.6$ kHz (i.e. $f/f_0=1$) at $\alpha =11.5$ kPa, (the top spectrum in Fig. 2a). These profiles were obtained by digitally filtering the measured signal using a narrow bandpass filter that was designed so that the central frequency would correspond to 24.6 kHz but the filter bandwidth would be wide enough not to cut the sidebands. We observe an amplitude-modulated signal with the modulation period close to that of the natural bubble cluster oscillations.

In the temporal profile in Fig. 3a, the amplitude modulation depth (the ratio of the modulation excursions to the amplitude of the unmodulated carrier wave) is smaller than 1. In some optical comb technologies, where direct photodetection of the optical pulses is used to produce an electronic signal that follows the amplitude modulation of the pulse train, low modulation depth could pose challenges for practical realisation^1^. However, this does not present a problem in our case because the frequencies of the electronic signal of AFC coincide with the frequencies of the driving pressure wave, which dramatically simplifies the characterisation of the comb.

The experimental time series is in good qualitative agreement with the calculated one shown in Fig. 3b. According to the discussion in Methods, the ultrasound energy absorption and scattering by the equivalent bubble are stronger than those by the bubble cluster. Because in the calculation the power of the driving ultrasound wave is the same as in the experiment, the oscillations of the equivalent bubble at its natural frequency are less energetic. Therefore, their interaction with the driving ultrasound waves should result in a weaker amplitude modulation. On the other hand, our asymptotic analysis of Eq. (1) demonstrates that in our model of the equivalent bubble the amplitude modulation also depends on the chosen initial conditions. Indeed, by varying in the calculation the initial radius of the bubble within ±5% we could achieve a nearly the same amplitude modulation as in the experiment. However, because of a complex interaction between the bubbles within the cluster^27,52,53^ and therefore essentially random initial conditions, it is challenging to establish the actual physical mechanism responsible for the discrepancy in the amplitude modulation in Fig. 3a,b.

**Figure 4:**
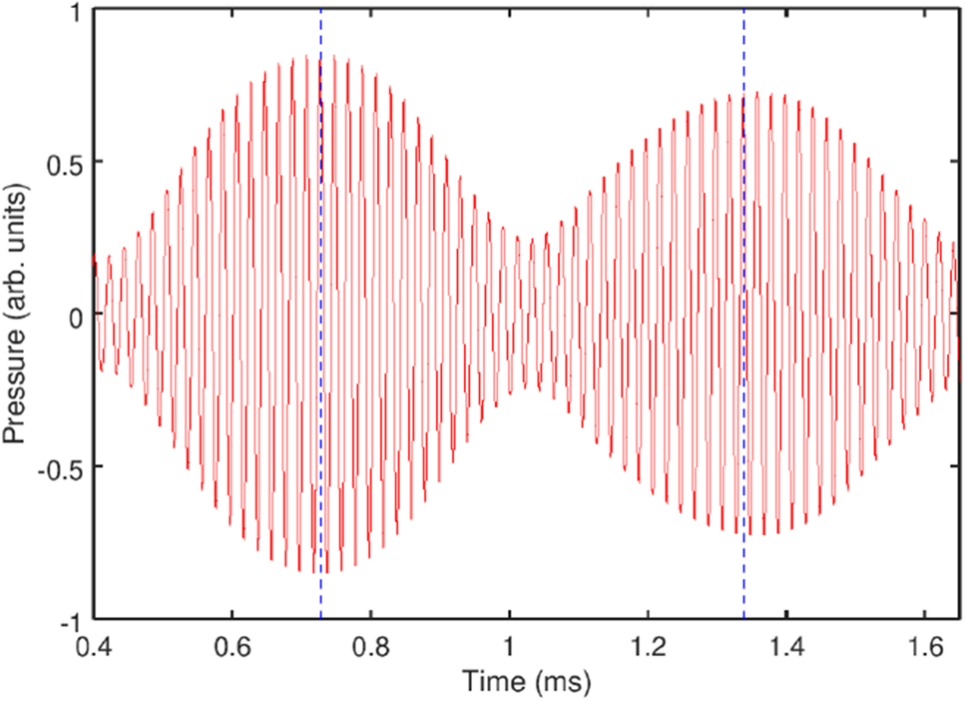
(**a**) Measured acoustic bubble cluster response corresponding to a sinusoidal driving pressure wave with the frequency $f_0=49.2$ kHz (twice the frequency in Fig. 3) and amplitude $\alpha =4.3$ kPa. The time between the vertical dashed lines is $\Delta T=1/f_{nat} \approx 0.6$ ms.

Next we focus on the experimental sideband peak structures at $f_0=49.2$ kHz (i.e. $f/f_0=2$) at $\alpha = 4.3$ kPa because it has three sidebands on each side. As shown in Fig. 4, the amplitude modulation gives rise to a train of pulses with the modulation period close to that of the natural bubble cluster oscillations.

We also note a stronger irregularity of the envelope shape in Fig. 4 compared with the profile in Fig. 3a, where a slight irregularity can only be seen in the close-up (inset in Fig. 3a). As discussed above, we attribute this difference to a translational motion of bubbles in the cluster^27,52,53^. The analysis of such a translation motion poses significant computational and experimental challenges^27^. However, a theoretical insight can be gained from the analysis of a system consisting of just two moving gas bubbles^52^, where it has been shown that stronger ultrasound forcing prevents bubble collision and coalescence thereby increasing the stability of their oscillations. On the other hand, similar to a single oscillating bubble^54–58^, the translational motion in bubble clusters should depend on the frequency and pressure of the driving wave. Thus, it is plausible to assume that driving the bubble cluster at a higher frequency but lower pressure, which is the case in Fig. 4, could compromise the stability of the cluster, which indeed has been predicted in Ref.^53^. Clearly, any deterioration of the cluster stability would adversely affect the coherence of its oscillations, which we believe is the reason for an imperfection of the envelope shape in Fig. 4. Admittedly, this effect is unfavourable for the generation of a frequency comb, and its further studies would be important from both the fundamental and practical points of view.

Finally, we note that the spectral peak structure of the AFCs demonstrated in this work should be stable with respect to the changes in the viscosity, density and surface tension of water due to the variations in the temperature and other environmental factors such as the salinity of water. Indeed, whereas the material parameters of water depend on ambient conditions and affect the natural bubble frequency in principle (see Eq. 4), such an influence on mm-size bubbles is negligible as discussed in the Model section.

## Discussion

AFC technique is an emerging metrological approach that benefits from technological maturity of optical frequency combs. It opens up opportunities for accurate measurements in various physical, chemical and biological systems in situations, where using light poses technical and fundamental limitations, for example, when precise underwater distance measurement is required^15^.

Whereas AFCs are expected to operate similarly to optical combs, there are a number of differences between these two techniques associated with the disparate frequencies of acoustic waves and light and mechanisms of the interaction of these waves with the medium^16^. This aspect presents numerous technological challenges that shape research efforts in the field^1,2^. We also note that frequency combs with a flat spectral intensity distribution are preferred in a number of applications^1,2^. In AFCs this has yet to be achieved. Moreover, as shown in refs.^14,16^, AFCs have the smaller number of spectral lines compared with optical combs. This feature has been identified as being important for a number of practical applications including phonon lasers^59,60^ and computing^61^.

In view of the above, AFCs should be compared with either Brillouin^62,63^ or opto-electronic combs^64^ that are known to also have a small number of lines and require special techniques for broadening their spectral ranges^65^. The same applies, although to a lesser degree, to Kerr optical combs that are generated using a cascade of optical four-wave mixing processes in a photonic microresonator^66^. Kerr combs may have just ten or so lines due to intrinsically low strength of nonlinear-optical effects^16,67^, which is in stark contrast to conventional optical combs based on mode-locked lasers and consisting of hundreds of lines^68^.

In good agreement with our numerical predictions, our experimental results demonstrate that a signal produced by gas bubbles oscillating in water has a frequency spectrum composed of equidistant peaks and is characterised by amplitude modulation at the bubble cluster natural frequency. These features are similar to those of typical optical frequency combs and thus they demonstrate the feasibility of the AFC generation by using gas bubble oscillations in a liquid. Although we were able to generate just a few comb lines, we found that their structure was similar to that produced in earlier works^14^. It should suffice for such practical applications as phonon lasers^59,60^ for the realisation of which an acoustic resonator filled with a liquid containing gas bubbles acting as the active medium was suggested^69^. In such a scheme, bubble oscillations have to be self-synchronised to resonate at a certain frequency, which can be achieved by appropriately tuning the AFC lines. Moreover, some techniques for spectral broadening of optical combs based on nonlinear-optical effects^64^ can be applied to increase the number of lines in a comb based on gas bubble oscillations because of the analogy between nonlinear optical and acoustical processes^16^. For example, as follows from our asymptotic analysis of Eq. (1), the number of lines in our AFC and their relative magnitude can be increased by simultaneously decreasing the frequency and increasing the pressure of the forcing ultrasound wave. As a result, one can obtain a spectrum where the sideband peaks around the fundamental and second harmonic frequency of the driving signal form a continuous comb structure. However, one should bear in mind that the frequency of the forcing has always to be higher than the natural oscillation frequency of the bubble cluster.

The so-generated AFCs should also find an application niche in the fields of underwater distance measurements and communication. However, their wider use is expected to be in the areas of biology and medicine, where there is a need for novel types of biomedical sensors. For example, the AFCs suggested here can be used to measure elastic properties of some biological tissues and living cells and sensing biochemical processes inside them via inducing elastic deformation in the proximity of an oscillating bubble^70,71^. Such a local mechanical deformation would affect the oscillation dynamics of the bubble^72^ and lead to detectable modifications of the sideband spectral structure of the comb. Thus, it should be possible to use bubbles oscillating in water contaminated with pathogens (e.g. bacteria) to obtain information about their presence and concentration that are required for choosing an adequate strategy for their removal^73^ or disinfection^74,75^.

Our AFCs can also be used to measure the resonance frequency of a bubble of unknown size^76,77^. Thus far, a number of bubble sizing techniques using two-frequency excitation have been employed^76,77^. There, two beams—a pump beam of variable frequency and an imaging beam of fixed frequency—are simultaneously used to scan across the expected resonance frequency of the bubble and to achieve the coupling between the two signal, when the bubble undergoes nonlinear oscillations at resonance. Using a frequency comb generated with just one driving wave will extend the capability of this technique because, from the technical point of view, only one ultrasound transducer needs to be employed.

## Data Availability

The datasets generated during and/or analysed during the current study are available from the corresponding author on reasonable request.
